# The Tacrolimus Concentration/Dose Ratio Does Not Predict Early Complications After Kidney Transplantation

**DOI:** 10.3389/ti.2023.11027

**Published:** 2023-05-09

**Authors:** Friedrich Alexander von Samson-Himmelstjerna, Maja Lucia Messtorff, Nassim Kakavand, Ute Eisenberger, Johannes Korth, Ulrich Lange, Benedikt Kolbrink, Leon Aldag, Tobias Schulze Dieckhoff, Thorsten Feldkamp, Ulrich Kunzendorf, Ana Harth, Kevin Schulte

**Affiliations:** ^1^ Department of Nephrology and Hypertension, University Hospital Schleswig-Holstein, Christian-Albrechts-University, Kiel, Germany; ^2^ Department of Nephrology, Essen University Hospital, Essen, Germany; ^3^ Department of Nephrology, Krankenhaus Köln-Merheim, Klinikum der Universität Witten/Herdecke, Cologne, Germany

**Keywords:** kidney transplantation, tacrolimus, acute graft rejection, C/D ratio, BKV nephritis

## Abstract

Early-on post kidney transplantation, there is a high risk of graft rejection and opportunistic viral infections. A low tacrolimus concentration/dose (C/D) ratio as a surrogate marker of fast tacrolimus metabolism has been established for risk stratification 3 months post-transplantation (M3). However, many adverse events occurring earlier might be missed, and stratification at 1 month post-transplantation (M1) has not been investigated. We retrospectively analyzed case data from 589 patients who had undergone kidney transplantation between 2011 and 2021 at three German transplant centers. Tacrolimus metabolism was estimated by use of the C/D ratio at M1, M3, M6, and M12. C/D ratios increased substantially during the year, particularly between M1 and M3. Many viral infections and most graft rejections occurred before M3. Neither at M1 nor at M3 was a low C/D ratio associated with susceptibility to BKV viremia or BKV nephritis. A low C/D ratio at M1 could not predict acute graft rejections or impaired kidney function, whereas at M3 it was significantly associated with subsequent rejections and impairment of kidney function. In summary, most rejections occur before M3, but a low C/D ratio at M1 does not identify patients at risk, limiting the predictive utility of this stratification approach.

## Graphical Abstract

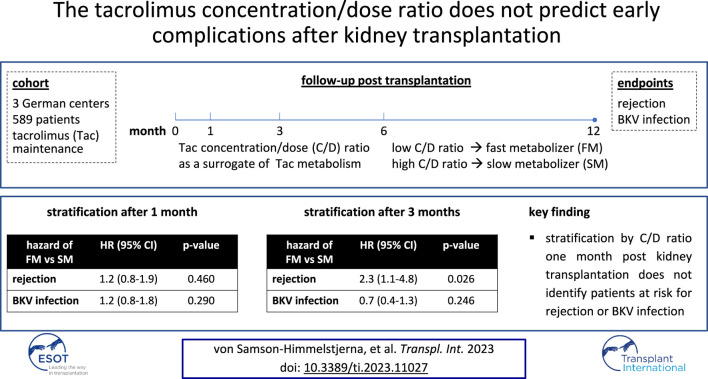

## Introduction

Kidney transplantation is the best long-term renal replacement therapy for quality of life and survival (1). Although advances in immunosuppression therapy have increased graft tolerance, acute and chronic rejections remain the greatest threat to graft survival (2). The calcineurin inhibitor tacrolimus is a mainstay of the most common combination of immunosuppressants to maintain graft tolerance (3). Tacrolimus is mainly metabolized by the hepatic enzymes CYP3A4 and CYP3A5, the activity of which can be influenced by several factors such as comedication, diet, and genetic polymorphisms (4). If not monitored closely, drug levels can therefore be inadequate. Low tacrolimus levels lead to insufficient immunosuppression with the risk of graft rejection, whereas high levels can have nephrotoxic effects and increase the risk of opportunistic infections (5). Infections with cytomegalovirus (CMV) and with the polyomavirus BK virus (BKV) are particularly common and are most frequent during the first month post-transplantation (6, 7). In immunocompromised patients, CMV can cause life-threatening organ diseases such as pneumonitis and colitis, whereas BKV causes BKV nephritis (BKVN) with potentially severe graft dysfunction (7, 8). Consequently, tailoring the right dose of tacrolimus to each patient is a balancing act.

The tacrolimus metabolism rate varies extensively between kidney graft recipients. A simple way of estimating the metabolism rate was proposed by Thölking et al., who suggested using the concentration/dose (C/D) ratio as a surrogate marker (9). The authors found an increased risk of acute graft rejection, impairment of kidney function, and incidence of BKVN in patients with a low C/D ratio, who were defined as fast metabolizers (9–12). In a French cohort, fast metabolism was associated with death-censored graft failure (13). The C/D ratio could therefore be used as a readily available risk stratification tool. Previous studies have used 3 months (M3) to 6 months (M6) post-transplantation as the time to divide patients into metabolizer groups (11–13). However, because many opportunistic infections and rejections occur prior to M3 and M6, the practical value of the C/D ratio may be higher if an earlier time for stratification such as 1 month post-transplantation (M1) was established. This would require a stable tacrolimus metabolism at M1, but unfortunately, the stability of the C/D ratio during the early phase post-transplantation has not been well studied thus far, and the available literature yields contradictory results: Jouve et al. report a highly stable metabolism between M3, M6, and 12 months post-transplantation (M12), while a smaller case-control study showed a tendency of increasing C/D ratios from M1 to M6 (10, 13). Therefore, we intended to answer the following questions in this retrospective, multicenter study.

i) How stable is the C/D ratio throughout the first year after kidney transplantation?
ii) Can early determination of the C/D ratio at M1 predict viral infections?
iii) Can early determination of the C/D ratio at M1 predict acute graft rejections?

## Patients and Methods

### Study Population

**FIGURE 1:**
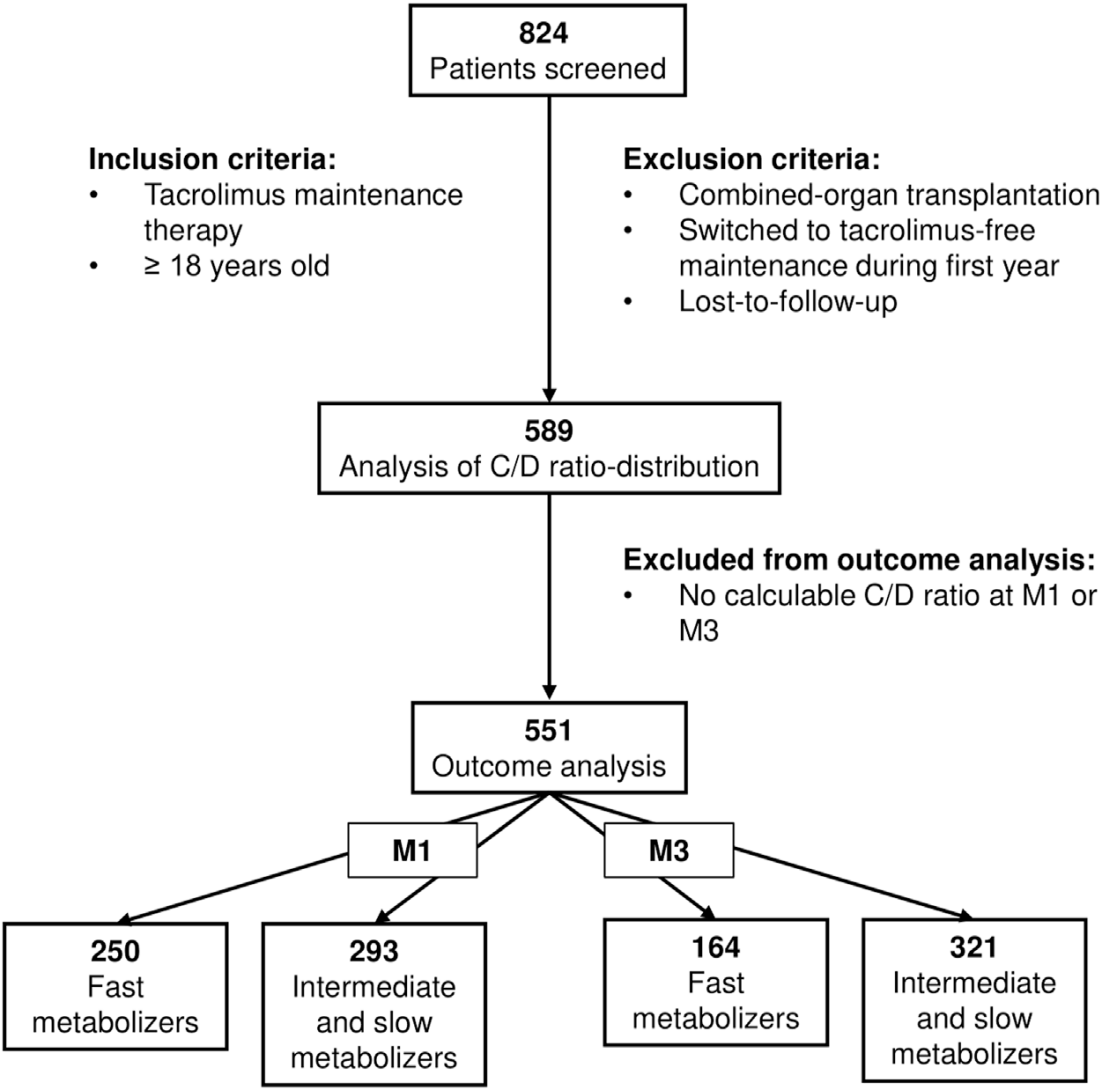
Flowchart of patient enrollment. Kidney transplant recipients (deceased or living donors) between 2011 and 2021 were screened. Patients with tacrolimus maintenance therapy throughout the first year post-transplantation were included for analysis of the C/D ratio (*n* = 589), of which 38 patients were excluded from subsequent outcome analyses because of absence of calculable C/D ratios at M1 or M3. C/D, concentration/dose; M1/3/6/12, 1/3/6/12 months post-transplantation.

We screened 824 patients who had received kidney transplants from deceased or living donors at the three participating centers in Kiel, Essen, and Cologne-Merheim between February 2011 and July 2021. The standard immunosuppressive protocol at these centers included a twice-daily immediate-release formulation of tacrolimus (8–12 ng/mL during the first month, then subsequently 5–8 ng/mL), mycophenolate mofetil or mycophenolic acid, and prednisone (tapered to and continued at 5 mg/d by M6). Patients lost-to-follow-up, patients with combined-organ transplantations such as pancreas-kidney transplantation, and patients who had been switched to tacrolimus-free immunosuppression during the first year post-transplantation were not included in the study. We included 589 adult patients for analysis of tacrolimus metabolism who had at least one calculable C/D ratio available at M1, M3, M6, or M12. The subsequent analyses of 1-year outcomes contained only 551 patients because 38 patients did not have a calculable C/D ratio at M1 or M3 (Figure 1).

### C/D Ratio

Similar to Thölking et al’s study, C/D ratios were determined by dividing the tacrolimus trough level by the total daily dose. A C/D ratio <1.05 ng/(mg*ml) was considered as suggestive of fast tacrolimus metabolism, a C/D ratio between 1.05 and 1.55 ng/(mg*ml) as suggestive of intermediate metabolism, and a ratio ≥1.55 ng/(mg*ml) as suggestive of slow metabolism (9).

### Outcomes

We defined BKV or CMV viremia as any infection with a detectable copy load in the plasma by using polymerase chain reaction (PCR). Severe BKV infection was defined as a copy load exceeding 100,000 U/mL. BKVN was defined as biopsy-proven BKVN. CMV organ infection was defined as a clinical diagnosis of CMV esophagitis, enteritis, encephalitis, hepatitis, pneumonitis, or retinitis. Acute graft rejections were defined as biopsy-proven acute graft rejections of any entity according to the most recent Banff classification at the respective time that required immunosuppressive treatment and/or plasmapheresis, including borderline rejections (14). The estimated glomerular filtration rate (eGFR) was calculated according to the Chronic Kidney Disease Epidemiology Collaboration (CKD-EPI) (15). Kidney failure was defined as the permanent necessity of renal replacement therapy or re-transplantation. Death was defined as all-cause mortality.

### Ethical Approval

This study was approved by the ethics committee of the Christian-Albrecht University of Kiel (D 429/18).

### Statistical Analysis

All statistical analyses were conducted using GraphPad Prism (version 5.0). Two-sided t-test was used for normally distributed linear variables, Mann-Whitney test was used for non-normally distributed linear variables, and chi-square test (95% confidence level) was used for categorical variables, respectively, when two groups were compared. One-way ANOVA with Bonferroni *post hoc* test was used for the comparison of multiple groups. Log-rank (Mantel-Cox) tests were used for analysis of Kaplan-Meier survival curves. All hazard ratios (HR) were calculated at a 95% confidence level. Values of probability (p) <0.05 were considered statistically significant.

## Results

### The C/D Ratio Increases Throughout the First Year

**FIGURE 2:**
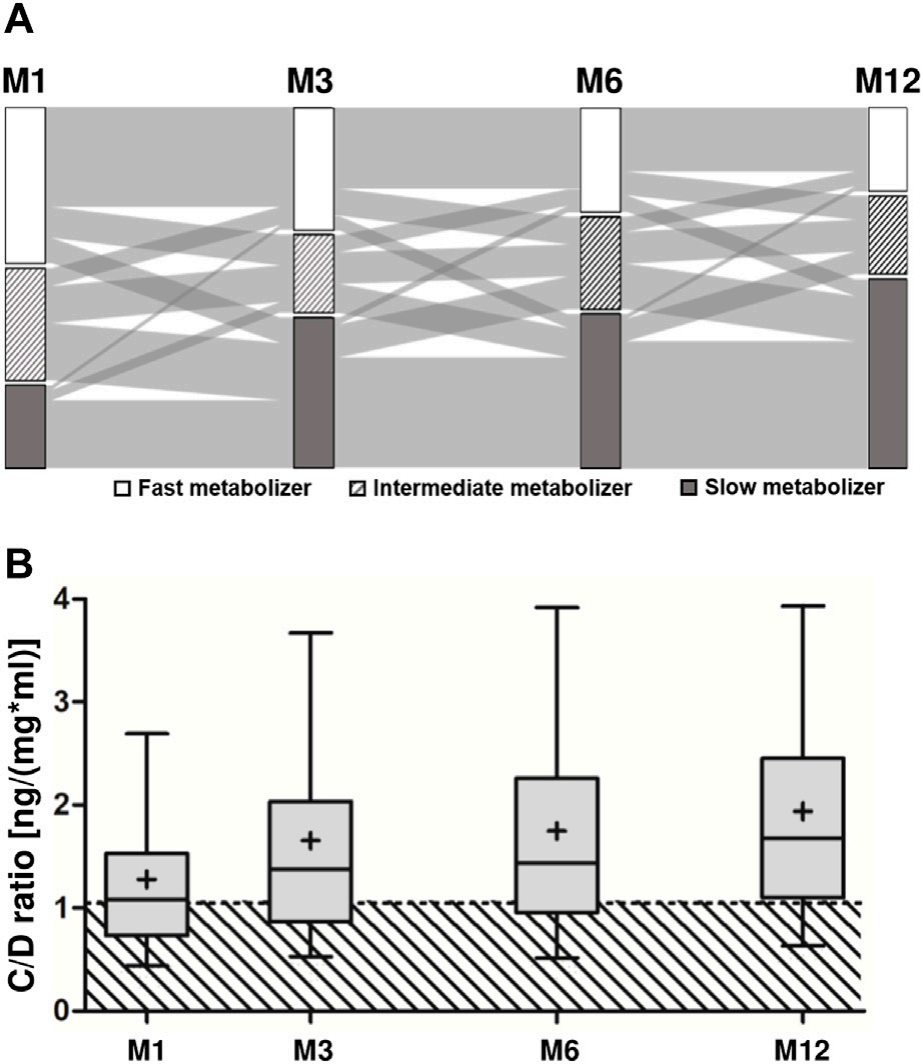
Dynamics of the C/D ratio in the first year post-transplantation. **(A)** Patients were stratified as fast (<1.05 ng/[mg*ml]), intermediate (1.05–1.54 ng/[mg*ml]), or slow (≥1.55 ng/[mg*ml]) metabolizers according to the C/D ratio at M1 (*n* = 543), M3 (*n* = 485), M6 (*n* = 458), and M12 (*n* = 540). **(B)** A box plot with interquartile ranges and 5%–95% whiskers at M1 to M12 (mean indicated by +) shows the median C/D ratio. The hatched area indicates C/D ratios below 1.05 ng/(mg*ml). C/D, concentration/dose; FM, fast metabolizer; M1/3/6/12, 1/3/6/12 months post-transplantation.

Baseline characteristics of the study group are shown in Table 1. Fast metabolizers stratified at M1 were significantly younger, had received more prednisone, and had lower tacrolimus trough levels than slow metabolizers. Stratifying at M3, there were no age differences between fast metabolizers and slow metabolizers, but fast metabolizers had lower trough levels. Regarding other baseline parameters, no significant differences were detected. Most patients had received a deceased donor transplantation with basiliximab induction therapy. Maintenance immunosuppression almost exclusively consisted of prednisone, mycophenolate, and tacrolimus. At M1, fast metabolizers represented the largest group (47%, Figure 2A). While 35% of the fast metabolizers at M1 underwent conversion to slow metabolism by M12, only 4% of slow metabolizers at M1 had developed fast metabolism by M12. The relative number of patients classified as fast metabolizers reduced quickly initially, from 47% at M1 to 35% at M3, but stabilized thereafter, declining to 29% and 23% at M6 and M12, respectively. Throughout the first year post-transplantation, the mean C/D ratio in the full cohort increased from 1.28 ng/(mg*ml) at M1 to 1.92 ng/(mg*ml) at M12 (Figures 2A,B).

**TABLE 1:** Baseline characteristics of patients included in the study.

Characteristic	Full cohort *n* = 551	M1			M3		
		FM *n* = 250	IM & SM *n* = 293	*p*	FM *n* = 164	IM & SM *n* = 321	*p*
Age mean (SD)	52.1 (±14.3)	49.9 (±14.6)	54.0 (±13.8)	***	49.4 (±15.3)	51.4 (±14.6)	ns
BMI (SD)	25.8 (±5.0)	25.5 (±5.1)	26.1 (±4.8)	ns	25.7 (±4.8)	25.9 (±5.1)	ns
Male	342 (62.1%)	146 (58.4%)	190 (64.8%)	ns	94 (57.3%)	205 (63.9%)	ns
Female	209 (37.9%)	104 (41.6%)	103 (35.2%)	ns	70 (42.7%)	116 (36.1%)	ns
Deceased donor graft	400 (72.6%)	177 (70.8%)	216 (73.7%)	ns	110 (67.1%)	242 (75.4%)	ns
Living donor graft	151 (27.4%)	73 (29.2%)	77 (26.3%)	ns	54 (32.9%)	79 (24.6%)	ns
First TX	514 (93.3%)	235 (94.0%)	272 (92.8%)	ns	151 (93.1%)	304 (94.7%)	ns
≥ Second TX	37 (6.7%)	15 (6%)	21 (7.2%)	ns	13 (7.1%)	17 (5.3%)	ns
Donor CMV+	278 (50.5%)	124 (49.7%)	151 (51.5%)	ns	79 (48.2%)	168 (52.3%)	ns
Recipient CMV+	329 (49.5%)	147 (58.8%)	178 (60.8%)	ns	97 (59.1%)	194 (60.4%)	ns
Induction therapy							
ATG	109 (19.8%)	52 (20.8%)	54 (18.4%)	ns	34 (20.7%)	61 (19.0%)	ns
Basiliximab	442 (90.2%)	198 (79.2%)	241 (82.3%)	ns	130 (79.3%)	260 (81.0%)	ns
Initial maintenance							
Tac, MMF, Predni	527 (95.6%)	242 (96.8%)	282 (96.2%)	ns	156 (95.1%)	309 (96.3%)	ns
Tac, Eve, Predni	21 (4.4%)	8 (3.2%)	11 (3.8%)	ns	5 (3%)	12 (3.7%)	ns
Maintenance at M12							
Tac, MMF, Predni	515 (93.5%)	235 (94.0%)	274 (93.5%)	ns	155 (94.5%)	299 (93.1%)	ns
Tac, Eve, Predni	31 (5.6%)	12 (4.8%)	19 (6.5%)	ns	9 (5.5%)	22 (6.9%)	ns
Prednisone (mg/d)							
M1	15.8 (±9.56)	17.1 (±10.8)	14.7 (±8.4)	**	16.27 (±10.9)	15.3 (±8.8)	ns
M3	8.4 (±4.3)	8.6 (±4.8)	8.2 (±3.9)	ns	8.9 (±5.4)	8.2 (±3.6)	ns
M6	6.6 (±6.9)	6.4 (±6.9)	6.7 (±7.0)	ns	6.1 (±2.2)	6.6 (±6.6)	ns
M12	5.7 (±4.4)	5.6 (±4)	5.8 (±4.8)	ns	5.9 (±5.6)	5.5 (±2.6)	ns
Tac dose (mg/d)							
M1	9.7 (±5.0)	13.1 (±4.6)	6.8 (±2.7)	****	13.3 (±5.1)	8.0 (±3.8)	****
M3	6.7 (±3.7)	9.0 (±3.7)	4.9 (±2.3)	****	10.5 (±3.3)	4.8 (±2.0)	****
M6	5.5 (±3.3)	7.2 (±3.7)	4.0 (±2.0)	****	8.4 (±3.5)	3.9 (±1.7)	****
M12	4.7 (±2.8)	6.0 (±3.1)	3.6 (±1.9)	****	7.1 (±3.1)	3.6 (±1.8)	****
Tac level (ng/mL)							
M1	9.6 (±3.1)	8.6 (±2.5)	10.4 (±3.3)	****	9.3 (±3.1)	9.7 (±3.0)	ns
M3	8.2 (±2.8)	8.3 (±2.3)	8.2 (±3.2)	ns	7.2 (±1.9)	8.8 (±3.0)	****
M6	7.0 (±2.1)	7.0 (±2.1)	7.0 (±2.1)	ns	7.0 (±2.2)	7.1 (±2.0)	ns
M12	6.7 (±2.0)	6.9 (±2.2)	6.6 (±1.8)	*	6.9 (±1.7)	6.7 (±2.1)	ns

Patients with calculable C/D ratios were stratified at M1 and M3. Significance as indicated: ns *p*-value ≥0.05, * *p*-value <0.05, ** *p*-value <0.01, *** *p*-value <0.001, ****p-value <0.0001. ATG, anti-thymocyte globulin; BMI, body mass index; CMV+, IgG positive for cytomegalovirus; Eve, everolimus; FM, fast metabolizer; IM, intermediate metabolizer; M1/3/6/12, 1/3/6/12 months post-transplantation, MMF, mycophenolate mofetil; Predni, prednisone; SD, standard deviation; SM, slow metabolizer; Tac, tacrolimus; TX, transplantation.

### Fast Metabolizer Status is Not Associated With Viral Infection

**FIGURE 3:**
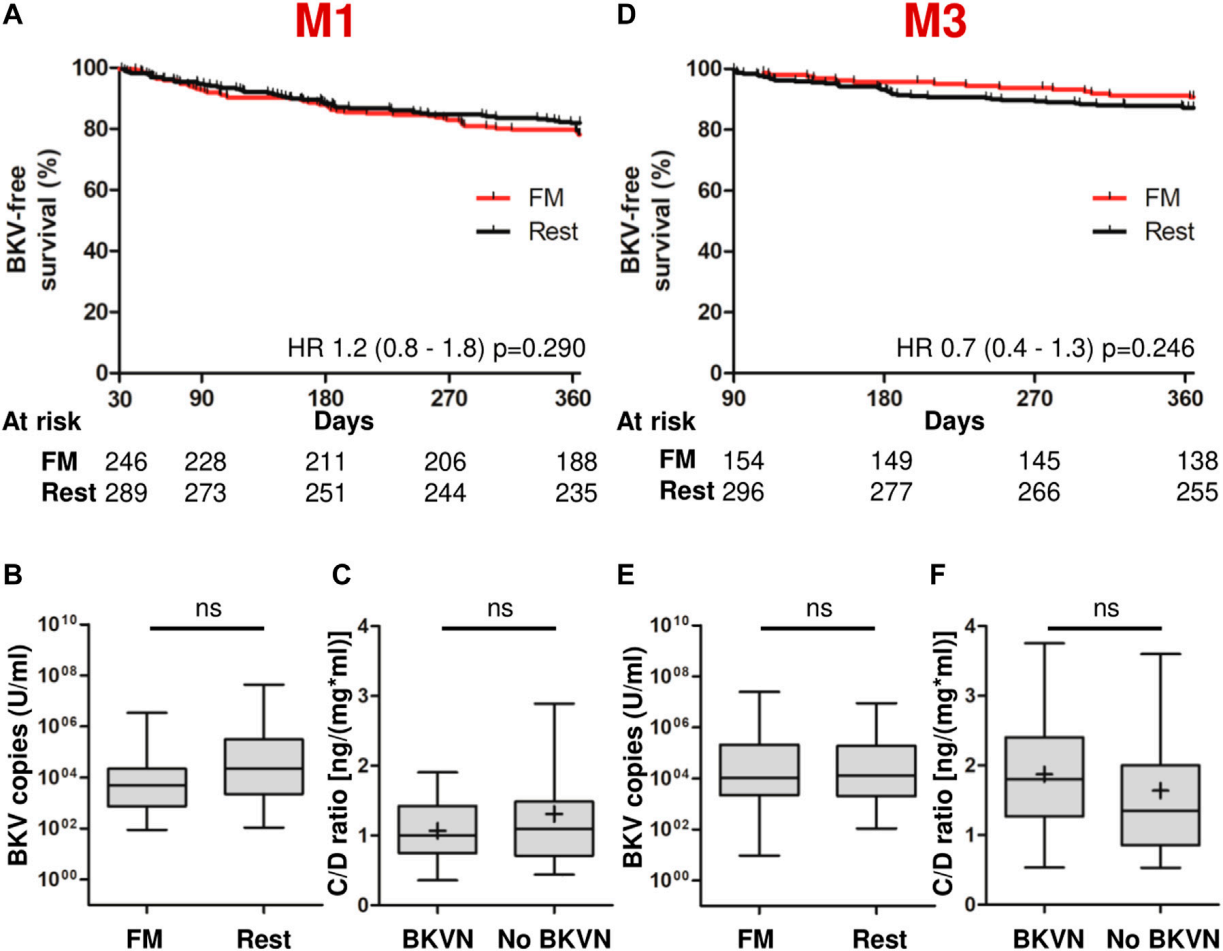
Fast tacrolimus metabolism does not predict BKV infections. Patients with infections before the respective stratification points (M1 or M3) were not included in the analyses. **(A)** Kaplan-Meier-viremia-free survival (death censored) of FMs and the rest of the cohort, as determined at M1. **(B)** A box plot with interquartile ranges and 5%–95% whiskers shows the viral BKV copy loads in the plasma in cases of viremia, as quantified by PCR. *n* = 53 for FM, *n* = 52 for the rest of the cohort. **(C)** A box plot with interquartile ranges and 5%–95% whiskers (mean indicated by +) shows median C/D ratios of patients with BKVN at M1 (*n* = 27) compared to those of patients without infection (*n* = 516). **(D)** Kaplan-Meier-viremia-free survival (death censored) of FMs and the rest of the cohort, as determined at M3. **(E)** Identical analysis as **(B)** in patients stratified at M3. *n* = 15 for FMs, *n* = 40 for the rest of the cohort. **(F)** Identical analysis as **(C)** was done to evaluate the C/D ratios at M3. *n* = 25 for BKVN and *n* = 458 for patients without BKVN. ns, not significant, *p-value <0.05, **p-value <0.01, ***p-value <0.001, ****p-value <0.0001. BKV, BK virus; BKVN, BKV nephritis; C/D, concentration/dose; FM, fast metabolizer; M1/3/6/12, 1/3/6/12 months post-transplantation.

Throughout the year, BKV viremia occurred in 20.1% of all patients and BKVN occurred in 4.9% (Table 2). CMV viremia was more frequent than BKV viremia, affecting 25.2% of the full cohort, but severe infection with impaired organ function occurred only in 2.2% (Table 2). Within the first 3 months post-transplantation, 35.1% of all BKV viremias and 43.9% of all CMV viremias had already been detected (Table 2 and Supplementary Figure S1). We determined the metabolizer status at M1 or M3 and disregarded patients with infections prior to these points, respectively, to assess a potential utility of the C/D ratio in predicting viral infections. Neither determination of fast metabolism at M1 (HR 1.2 [95% CI 0.8–1.8], *p* = 0.290) nor at M3 (HR 0.7 [95% CI 0.4–1.3], *p* = 0.246) was associated with a successively increased occurrence of BKV viremia when compared with the rest of the cohort (Figures 3A,D). Similarly, subsequent CMV viremia was neither associated with fast metabolism at M1 (HR 1.1 [95% CI 0.8–1.6], *p* = 0.549) nor at M3 (HR 0.8 [95% CI 0.5–1.3], *p* = 0.329; Supplementary Figures S2A,D). Metabolism status was not significantly associated with BKV or CMV plasma copy loads in patients who developed viremia (Figures 3B,E and Supplementary Figures S2B,E). Patients with severe infection such as BKVN or CMV organ infection did not have significantly different C/D ratios than patients without severe infection (Figures 3C,F and Supplementary Figures S2C,F), and neither BKVN nor CMV organ infection was associated with fast metabolism (Table 2). In a further analysis independent of the pre-specified time points, we observed the C/D ratio and the time-to-maximum-copy-load for cases with severe BKV infection (maximum copy load exceeding 100,000 U/mL). Since the time-to-maximum-copy-load was approximately 6 months (mean 168 days) in that group, we chose the C/D ratios of all patients without BKV viremia at M6 for comparison. Patients with severe BKV infection did not have significantly lower C/D ratios than patients without BKV infection (1.55 vs. 1.46; *p* = 0.801; Table 3).

**TABLE 2:** One-year outcomes for patients stratified at M1 or M3.

Characteristic	Full cohort *n* = 551	M1			M3		
		FM *n* = 250	IM & SM *n* = 293	*p*	FM *n* = 164	IM & SM *n* = 321	*p*
Graft failure	10 (1.8%)	4 (1.6%)	6 (2.0%)	ns	1 (0.6%)	5 (1.6%)	ns
Death	5 (0.9%)	2 (0.8%)	2 (0.7%)	ns	2 (1.2%)	3 (0.9%)	ns
BKVN	27 (4.9%)	10 (4.0%)	17 (5.8%)	ns	5 (3.0%)	20 (6.2%)	ns
BKV viremia	111 (20.1%)	56 (22.4%)	55 (18.8%)	ns	25 (15.2%)	64 (19.9%)	ns
Before M1	6 (5.4%)	3 (5.4%)	3 (5.5%)		0 (0.0%)	5 (7.8%)	
M3	39 (35.1%)	21 (37.5%)	18 (32.7%)		10 (40.0%)	24 (37.5%)	
M6	77 (69.4%)	38 (67.9%)	39 (70.1%)		15 (60.0%)	42 (65.6%)	
M12	111 (100.0%)	56 (100.0%)	55 (100.0%)		25 (100.0%)	64 (100.0%)	
CMV organ infection	12 (2.2%)	5 (2.0%)	7 (2.4%)	ns	2 (1.2%)	8 (2.5%)	ns
CMV viremia	139 (25.2%)	66 (26.4%)	73 (24.9%)	ns	41 (25.0%)	83 (25.9%)	ns
Before M1	24 (17.3%)	10 (15.2%)	14 (19.2%)		7 (17.1%)	14 (16.9%)	
M3	61 (43.9%)	29 (44.0%)	32 (43.8%)		21 (51.2%)	32 (38.6%)	
M6	89 (64.0%)	43 (65.2%)	46 (63.0%)		28 (68.3%)	50 (60.2%)	
M12	139 (100.0%)	66 (100.0%)	73 (100.0%)		41 (100.0%)	83 (100.0%)	
BPAR	172 (31.2%)	91 (36.4%)	79 (27.0%)	*	72 (43.9%)	83 (25.9%)	****
Before M1	96 (55.8%)	55 (60.4%)	40 (50.6%)		44 (61.1%)	43 (51.8%)	
M3	131 (76.2%)	68 (74.7%)	61 (77.2%)		56 (77.8%)	64 (77.1%)	
M6	156 (90.7%)	83 (91.2%)	71 (89.9%)		66 (91.7%)	75 (90.3%)	
M12	172 (100.0%)	91 (100.0%)	79 (100.0%)		72 (100.0%)	83 (100.0%)	
eGFR (mean ± SD)							
M1	44.5 (±16.8)	44.9 (±17.6)	44.2 (±16.0)	ns	43.3 (±16.7)	44.8 (±17.0)	ns
M3	44.4 (±16.4)	45.1 (±17.4)	43.8 (±15.6)	ns	44.2 (±16.9)	44.5 (±16.2)	ns
M6	46.1 (±16.4)	44.8 (±16.5)	47.2 (±16.4)	ns	43.1 (±16.4)	47.3 (±16.0)	*
M12	48.7 (±19.1)	47.8 (±22.1)	49.2 (±16.1)	ns	45.4 (±18.9)	50.6 (±19.3)	**

All patients with calculable C/D ratios at the respective times were included in this analysis. Significance as indicated: ns *p*-value ≥0.05, * *p*-value <0.05, ** *p*-value <0.01, *** *p*-value <0.001, **** *p*-value <0.0001. BKV, BK virus; BKVN, BKV nephritis; BPAR, biopsy-proven acute graft rejection; CMV, cytomegalovirus; eGFR, estimated glomerular filtration rate (in mL/min/1.73 m^2^); FM, fast metabolizer; IM, intermediate metabolizer; M1/3/6/12, 1/3/6/12 months post-transplantation; ns, not significant; SD, standard deviation; SM, slow metabolizer.

**TABLE 3:** C/D ratios in patients with severe vs. no BKV infection.

BKV status	*n*	Time-to-maximum-copy-load in days (mean ± SD)	C/D-ratio (median [IQR])	Mann-Whitney test (*p*-value)
Severe BKV infection	32	168 ± 101	1.55 (1.19–2.08)	0.801
BKV negative	345	190 ± 36	1.46 (0.97–2.23)	

Severe BKV infection was defined as a copy load exceeding 100,000 U/mL. BKV negative patients with available C/D ratios 6 months post-transplantation served as controls. BKV, BK virus; IQR, interquartile range; SD, standard deviation.

### Very Early Determination of the C/D Ratio Cannot Predict Graft Rejections

**FIGURE 4:**
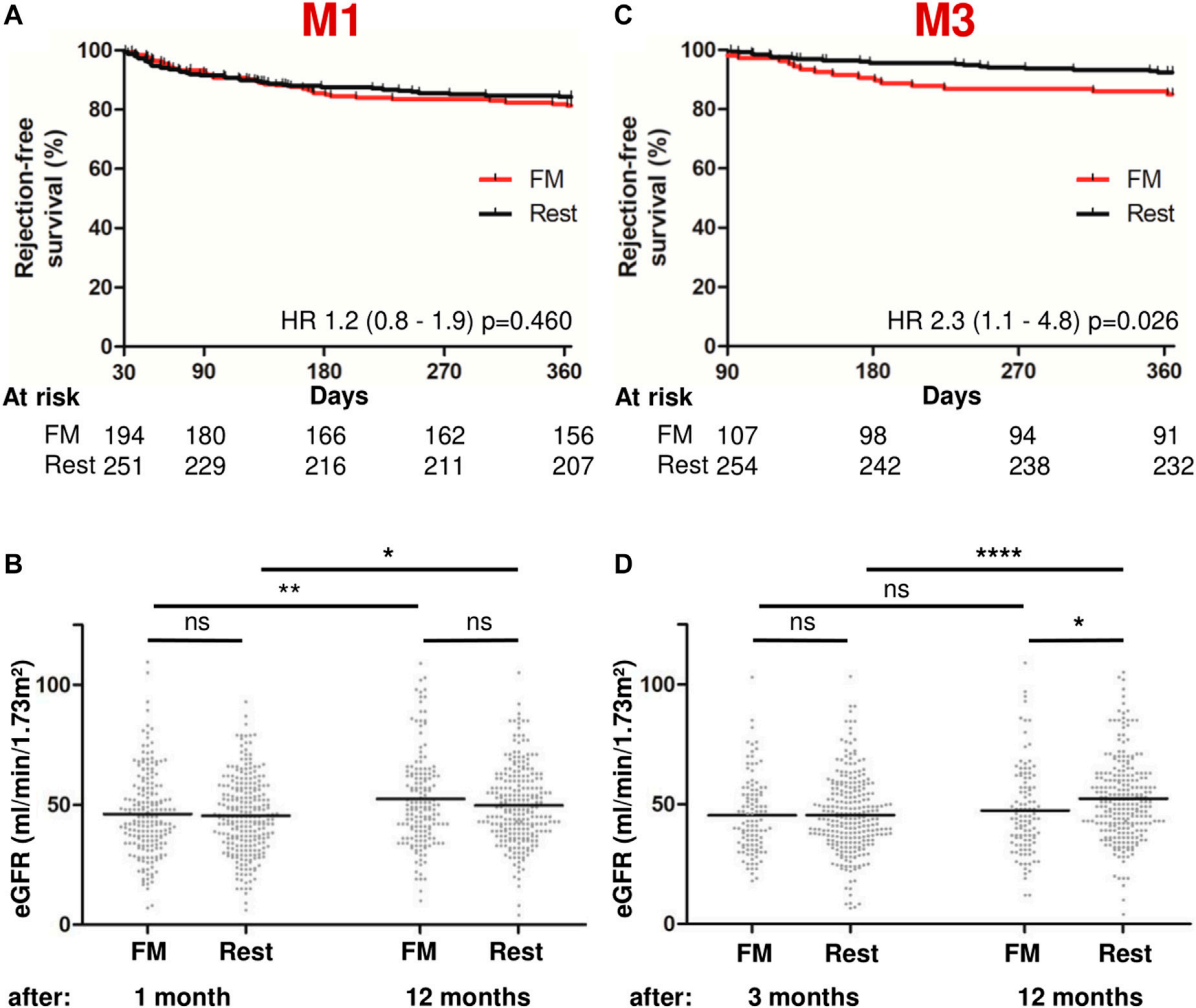
Fast metabolism at M3, but not at M1, predicts acute graft rejections and impaired graft function. Patients with rejections before the respective points of stratification (M1 or M3) were not included in the analyses. **(A)** Kaplan-Meier-rejection-free survival (death-censored) between the FM group and the rest of the cohort at M1. **(B)** Scatter plots with means showing eGFR at M1 and M12. **(C)** Kaplan-Meier-rejection-free survival (death-censored) between the FM group and the rest of the cohort at M3. **(D)** Scatter plots with means showing eGFR at M3 and M12. ns, not significant, *p-value <0.05, **p-value <0.01, ***p-value <0.001, ****p-value <0.0001. C/D, concentration/dose; eGFR, estimated glomerular filtration rate; FM, fast metabolizer; M1/3/6/12, 1/3/6/12 months post-transplantation.

We subsequently analyzed the association of acute graft rejections and kidney function with the C/D ratio. In the first-year post-transplantation, acute biopsy-proven graft rejections that required treatment ensued in 31.2%, 76.2% of which occurred during the first 3 months post-transplantation, but only few patients permanently lost their grafts (1.8%) or died (0.9%; Table 2). The kidney function in the full cohort, as indicated by the eGFR, was similar at M1 and M3, but had improved by M6 and M12 (Table 2). To assess the utility of the C/D ratio in predicting acute rejections, we determined fast metabolizer status at M1 and excluded all cases with rejections earlier than M1 (Figure 4A). Fast metabolism at M1 was not associated with an increased risk of subsequent episodes of graft rejection (HR 1.2 [95% CI 0.8–1.9], *p* = 0.460). Additionally, the eGFR of these fast metabolizers neither differed significantly from the rest of the cohort, nor did fast metabolizers show impaired graft development, as the eGFR in both groups improved significantly between M1 and M12 (46.2–52.4 mL/min/1.73 m^2^ and 45.4–49.7 mL/min/1.73 m^2^ in fast metabolizers and the rest of the cohort, respectively; Figure 4B). Contrarily, when patients were stratified by tacrolimus metabolism status at M3, fast metabolizers had a hazard ratio of 2.3 (95% CI 1.1–4.8, *p* = 0.026) for acute graft rejection between M3 and M12 as compared to patients with a slower tacrolimus metabolism (Figure 4C). In these fast-metabolizing patients, the eGFR increased slightly but non-significantly between M3 and M12 (45.4 and 47.4 mL/min/1.73 m^2^, respectively), whereas the eGFR of the more slowly metabolizing patients improved considerably from 45.4 at M3 to 52.4 mL/min/m^2^ at M12 (Figure 4D).

## Discussion

In this retrospective multicenter cohort study, we evaluated the tacrolimus C/D ratio of kidney graft recipients throughout the first-year post-transplantation and assessed whether earlier estimation of their metabolizer status than previously established could identify patients at risk for viral infection and graft rejection. We found that particularly during the early months after transplantation, the C/D ratio was unstable, and that it was not associated with opportunistic viral infections. Using the C/D ratio as a surrogate of metabolizer status 1 month post-transplantation did not identify patients at risk, but a low C/D ratio after 3 months predicted subsequent acute rejections and impaired kidney function.

The clinical purpose of determining the C/D ratio is to predict harmful events such as opportunistic viral infections or graft rejections. This could potentially enable transplant nephrologists to pre-emptively adjust immunosuppressive medication and more tightly monitor patients at risk. However, for such a preventative approach, the C/D ratio needs to be determined at the earliest opportunity, because most infections and rejections occur early after transplantation (5–7). Since tacrolimus metabolism is affected by co-medications such as prednisone, the dosing of which is subject to extensive changes during the first weeks post-transplantation, the C/D ratio may not be reliably estimated immediately after transplantation. Prednisone is known to induce metabolism of tacrolimus, and an increase of the C/D ratio would be expected as prednisone is tapered post-transplantation (16). Data from our cohort support this assumption, as only half of the fast metabolizers at M1 maintained their metabolism rate until M3. Thereafter, metabolism continued to reduce subsequently, but much less dynamically, and therefore, M3 appears to be the earliest reliable point at which metabolism rates should be determined.

However, our data also show that at M3, a substantial number of viral infections and acute graft rejections had already occurred, which severely limits the utility of the C/D ratio at M3. The evidence from other studies regarding the value of the C/D ratio earlier than at M3 is scarce, and previous large trials have not evaluated whether stratification of patients at a much earlier point would predict outcomes (9–12, 17). These questions were quite clearly answered in our cohort of patients: A low C/D ratio at M1 did not identify patients at risk for viral infection, acute rejection, or impaired graft function. Based on our data, the substantive increase in the C/D ratio between M1 and M3 in many patients rendered stratification at M1 unfeasible for risk prediction.

Fast metabolizers at M3 had significantly lower tacrolimus levels than slower metabolizers, which implies that fast metabolizers were consequently exposed to less immunosuppression. This might explain why we could confirm the previously reported association of fast metabolism at M3 with graft rejections and impaired kidney function (11, 12, 17), but not that with increased susceptibility to viral infection despite some studies reporting the opposite (9, 10).

Our study has some limitations. It should be kept in mind that we did not directly measure the tacrolimus metabolism, but rather used the C/D ratio as a surrogate marker. While this limits our ability to correlate the actual tacrolimus metabolism with clinical outcomes, it is a pragmatic approach because of its simplicity and clinical transferability. As this is a retrospective study, we were unable to draw causal assumptions from our findings and could only generate hypotheses. The study was underpowered to detect small differences of rarely occurring outcomes such as BKVN or CMV organ infections. However, the cohort size of nearly 600 patients from three transplant centers is one of the largest to evaluate the impact of tacrolimus metabolism on clinical outcomes, and sufficient power was present to detect differences in the primary outcomes. Additionally, as we were primarily interested in short-term complications, the follow-up period of 1 year was relatively short.

In conclusion, our study confirmed the C/D ratio as a pragmatic tool to identify patients at risk for graft rejections and sub-optimal development of kidney function after transplantation. Unfortunately, the C/D ratio only appears useful from M3 onwards, and many early complications can therefore not be addressed. Investigating whether fast metabolizers could benefit from switching an immediate-release to a prolonged-release formulation of tacrolimus, which allows more steady tacrolimus levels throughout the day, remains reasonable. In this respect, in a recent *post hoc* analysis of a randomized-controlled phase III trial, Suwelack et al. assessed if fast metabolizers, as determined at M1, had better outcomes when administered prolonged-release tacrolimus (18). Although rejections were less frequent in the prolonged-release group than in the immediate-release group, this difference was not statistically significant. Based on the findings of our study, we hypothesize that the authors’ timing of stratification at M1 was premature, and that stratification at M3 might have produced different results. Therefore, our findings provide a ground for the re-evaluation of efficacy of prolonged-release tacrolimus to prevent graft rejections in patients with fast metabolism. An alternative to tacrolimus maintenance was introduced with the co-stimulation inhibitor belatacept, which, compared to calcineurin inhibitors, improves long-term graft function (19–21). This benefit is limited by increased rejection rates in patients treated with belatacept compared to tacrolimus (21, 22). However, considering the results of this study and previous reports, patients with fast tacrolimus metabolism at M3 may have a fairly similar rejection risk as patients treated with belatacept. Consequently, the current restraints that prevent nephrologists from broadly prescribing belatacept to more patients might not apply to this patient group. Thus, a switch from tacrolimus to belatacept in fast metabolizers at M3 could be explored as a novel therapeutic concept.

## Data Availability

The raw data supporting the conclusion of this article will be made available by the authors, without undue reservation.

## Supplementary Material

Supplementary Figure S1 BKV viremia, CMV viremia, and acute graft rejections throughout the first year post-transplantation. This figure includes all patients from the cohort (*n* = 551). **(A–C)** show the Kaplan-Meier-event-free survival (death censored). Dashed lines indicate follow-ups at M1 after 30 days, M3 after 90 days, and M6 after 180 days. Follow-up ended at M12 after 365 days. Abbreviations: BKV, BK virus; CMV, cytomegalovirus; M1/3/6/12 = 1/3/6/12 months post-transplantation.

Supplementary Figure S2 Fast tacrolimus metabolism does not predict CMV infections. Patients with infections before the respective stratification points (M1 or M3) were not included in the analyses. **(A)** Kaplan-Meier-viremia-free survival (death censored) of FM and the rest of the cohort at M1. **(B)** A box plot with interquartile ranges and 5%–95% whiskers shows the viral CMV copy loads in the plasma in cases of viremia, as quantified by PCR. *n* = 56 for FM, *n* = 59 for rest. **(C)** A box plot with interquartile ranges and 5%–95% whiskers (mean indicated by +) shows median C/D ratios at M1 in patients with CMV organ infection (*n* = 12) compared to those of patients without infection (*n* = 531). **(D)** Kaplan-Meier-viremia-free survival (death censored) of FMs and the rest of the cohort, as determined at M3. **(E)** Identical analysis as **(B)** in patients stratified at M3. *n* = 20 for FM, *n* = 51 for rest of the cohort. **(F)** Identical analysis as **(C)** evaluating the C/D ratios at M3. *n* = 10 for CMV organ infection and *n* = 473 for no CMV organ infection. ns, not significant, **p* value < 0.05, ***p*-value < 0.01, ****p*-value < 0.001, *****p*-value < 0.0001. CMV, cytomegalovirus; C/D, concentration/dose; FM, fast metabolizer; M1/3/6/12, 1/3/6/12 months post-transplantation.

## Abbreviations

ANOVA, analysis of variance; ATG, anti-thymocyte globulin; BKV, BK virus; BKVN, BKV nephritis; BMI, body-mass-index; C/D, concentration/dose; CI, confidence interval; CKD-EPI, chronic kidney disease epidemiology collaboration; CMV, cytomegalovirus; eGFR, estimated glomerular filtration rate; Eve, everolimus; FM, fast metabolizer; HR, hazard ratio; IM, intermediate metabolizer; IR, immediate-release; FM, fast metabolizer; M1/3/6/12, 1/3/6/12 months post-transplantation; MMF, mycophenolate mofetil; ns, not significant; p, value of probability; PCR, polymerase chain reaction; PR, prolonged-release; Predni, prednisone; SD, standard deviation; SM, slow metabolizer; Tac, tacrolimus; TX, transplantation.
