# Supplementary figures and images for: The Tacrolimus Concentration/Dose Ratio Does Not Predict Early Complications After Kidney Transplantation
Source: Transpl Int. 2023 May 9;36:11027. doi: 10.3389/ti.2023.11027 (PMC10203205; doi:10.3389/ti.2023.11027)

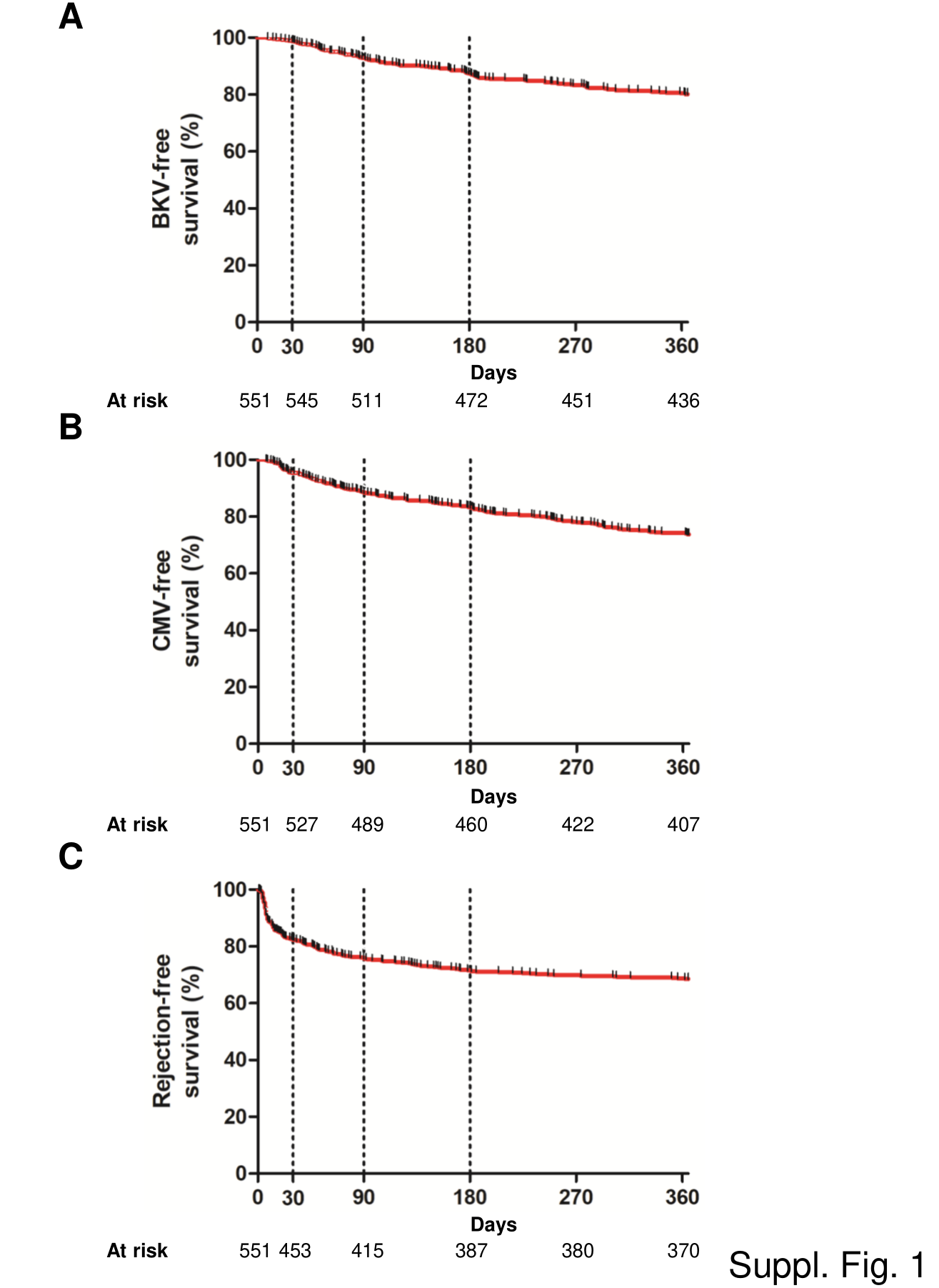

Supplement: Supplementary file 1 [file Image1.tiff]

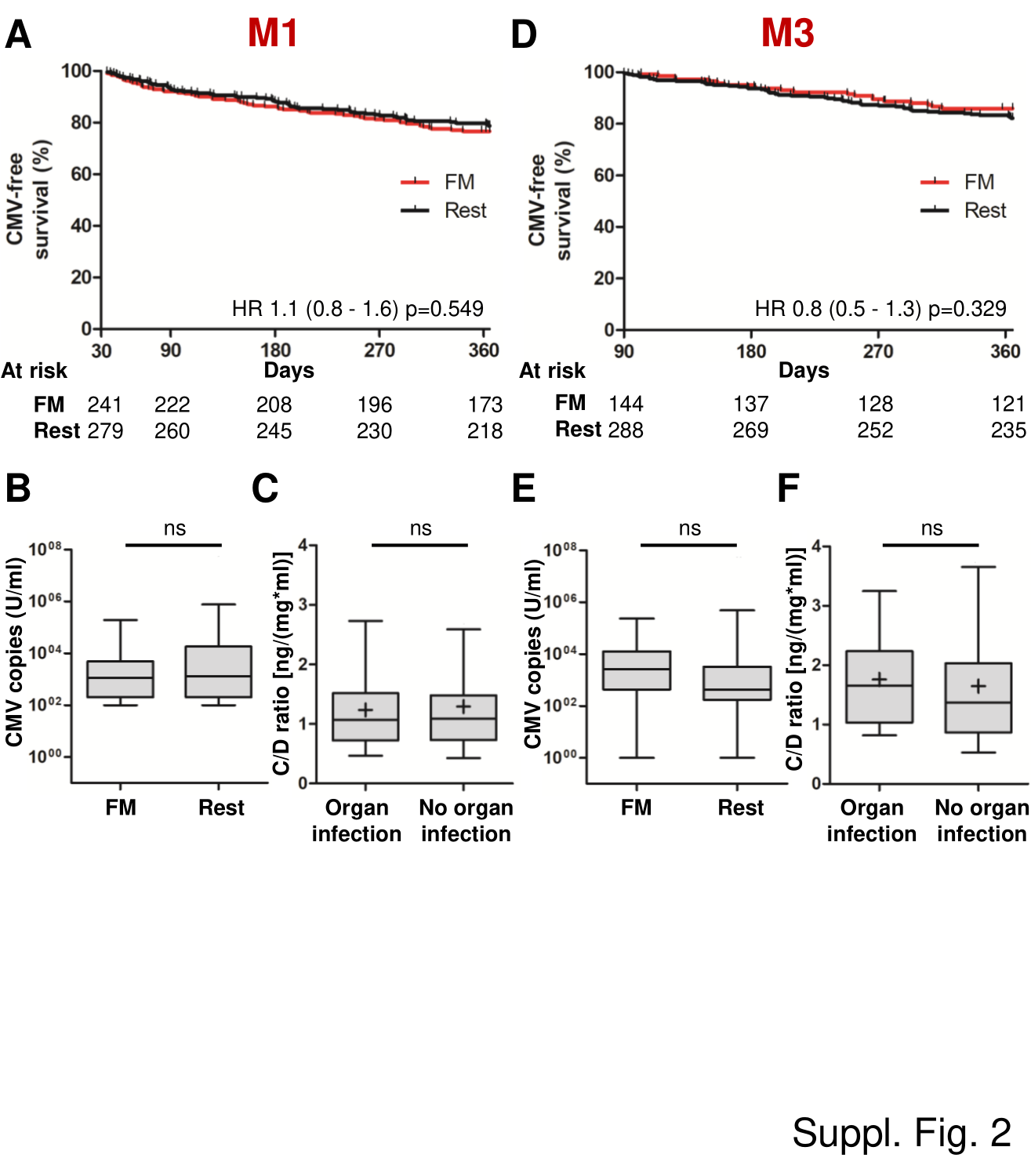

Supplement: Supplementary file 2 [file Image2.tiff]
